# Differentiating Leukostasis From COVID-19 Pneumonia: Clinical and Radiological Perspectives for the Right Decision-Making

**DOI:** 10.7759/cureus.56708

**Published:** 2024-03-22

**Authors:** Nouama Bouanani, Ibtihal Abouchabaka

**Affiliations:** 1 Clinical Hematology, Cheikh Khalifa International University Hospital, Casablanca, MAR; 2 Hematology, Mohammed VI University of Health Sciences, UM6SS, Casablanca, MAR

**Keywords:** patient management, differentiation, pneumonia, covid-19, leukostasis

## Abstract

Leukostasis is a non-infectious complication of acute leukemia. Pathophysiologically, it is characterized by an accumulation of blasts in the pulmonary capillaries. Clinically, this syndrome of hyperleukocytosis or leucocytosis leads to pulmonary and/or neurological lesions. This is why it must be treated urgently to prevent it from progressing to acute respiratory distress. In 2020, the World Health Organization (WHO) declared a pandemic caused by a novel coronavirus called SARS-CoV-2, which may cause respiratory distress or other clinical, biological, and radiological signs that some may confuse with those of leukostasis.

In this context, we present a compelling case study of a 64-year-old patient with no notable pathological antecedents and not vaccinated against COVID-19, who presented with acute respiratory distress. The purpose of our article is to succeed in differentiating between the two pneumopathies, thus making it possible to orient the doctor toward the right decision-making. As known, early recognition enables timely interventions, reducing disease progression and associated complications while also preventing unnecessary treatments, optimizing healthcare resources, and advancing medical knowledge for improved patient care in both acute leukemia and COVID-19 management.

## Introduction

Acute leukemia is a hematological malignancy that can manifest in two distinct forms: pancytopenia and hyperleukocytic [1]. Patients affected by this disease may develop pulmonary complications during the acute phase, which can be either infectious or non-infectious in nature. Among the non-infectious complications, three specific pulmonary manifestations have been identified: pulmonary leukostasis, pulmonary infiltration, and lysis pneumopathy. Pulmonary leukostasis [1], found in 5% to 20% of cases, is a critical non-infectious complication of acute leukemia, characterized by the accumulation of leukemia blasts within the pulmonary capillaries [2]. Urgent treatment is required to prevent its progression into acute respiratory distress.

In the backdrop of this, the year 2020 witnessed the World Health Organization (WHO) declaring a pandemic caused by a novel coronavirus named SARS-CoV-2, responsible for causing cases of respiratory distress worldwide [3,4]. In this context, we present a compelling case study of a 64-year-old patient with no notable pathological antecedents and not vaccinated against coronavirus disease 2019 (COVID-19), who presented with acute respiratory distress. Owing to the clinical and para-clinical similarities between leukostasis and SARS-CoV-2-induced respiratory distress, distinguishing between the two conditions can pose challenges.

The primary objective of this article is to elucidate the key clinical, radiological, and laboratory findings that can aid in the accurate diagnosis and early management of patients presenting with acute respiratory distress. By exploring the differentiating factors between leukostasis and SARS-CoV-2 pneumonia, we aim to provide valuable insights to physicians, facilitating timely and appropriate interventions for improved patient outcomes.

## Case presentation

In this case, we describe a 64-year-old patient with no notable pathological antecedents who had not received the COVID-19 vaccine and presented with a two-week history of asthenia. The patient was admitted to the emergency department due to respiratory distress. There were no notable personal or family pathological antecedents. During the general examination, the patient was conscious and well-oriented in time and space, with a performance status of 3. He was hemodynamically stable (blood pressure at 110/70 mm Hg and heart rate at 70 bpm) but had a fever of 39°C and was breathing rapidly at 28 cycles per minute, with oxygen saturation at 84% in ambient air. Physical examination revealed bilateral cracklings in the lungs and painless bilateral laterocervical lymphadenopathies measuring 2 cm. Additionally, splenomegaly was observed, with an increase of 2 cm in size. Mucocutaneous examination showed pale mucous membranes and skin with diffuse purpuric spots.

**Figure 1:**
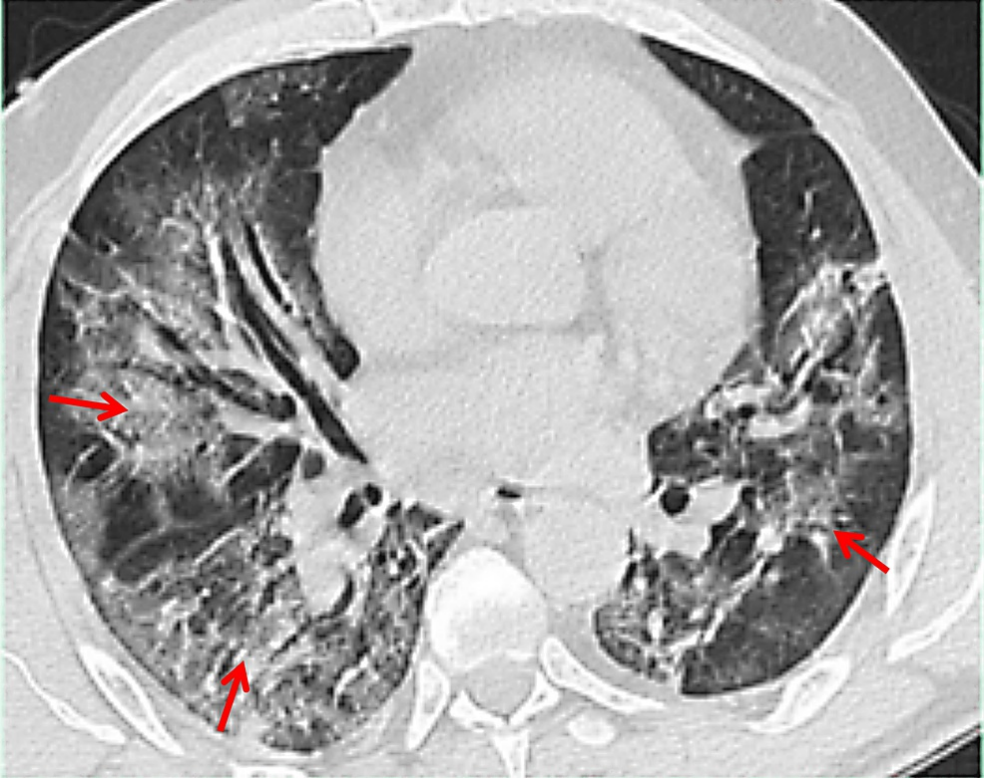
Axial chest CT scan with a parenchymal window shows a large ground glass and thickening of the bronchovascular axes (the arrows indicate some areas of lesions).

A chest computed tomography with a parenchymal window revealed bilateral interstitial and alveolar infiltration with a ground glass appearance (Figure 1), classified as coronavirus disease 2019 (COVID-19) Reporting and Data System (CO-RADS 4).

The CO-RADS 4 is specified as a high level of suspicion for pulmonary involvement of COVID-19 (Table 1).

**Table 1:** CO-RADS score. References: [5,6]. RT-PCR: reverse transcription polymerase chain reaction; CO-RADS: coronavirus disease 2019 (COVID-19) Reporting and Data System.

CO-RADS score		
CO-RADS classes	Level of suspicion for pulmonary involvement of COVID-19	CT findings
CO-RADS 1	No	Normal
CO-RADS 2	Low	Typical for infections other than COVID-19
CO-RADS 3	Indeterminate	It could be COVID-19 but it could also be another disease
CO-RADS 4	High	Suspicious for COVID-19
CO-RADS 5	Very high	Typical for COVID-19
CO-RADS 6	Proven (PCR)	RT-PCR positive for SARS-COV 2

The patient underwent multiple screenings, including a respiratory viral panel, antigenic tests, and RT-PCR SARS-CoV-2 tests on nasopharyngeal swabs, all of which yielded negative results. Laboratory studies revealed several abnormalities, including normochromic normocytic anemia with a hemoglobin level of 7 g/dl, thrombocytopenia (TP) with platelet count at 10,000/mm^3^, and significant hyperleukocytosis with a white blood cell count of 145,000/mm^3^ and 55% blasts in the periphery. Additionally, the patient had a fibrinogen level of 1.5 g/l, TP at 50%, normal tricyclic antidepressants (TCAs), D-dimers at 1000 ng/ml, lactate dehydrogenase (LDH) at 800 U/L, uric acid at 120 mg/L, serum potassium at 4 mmol/L, CRP at 200 mg/L, and a negative procalcitonin. The infectious assessment did not isolate any germs.

To evaluate the possibility of COVID-19 involvement, the CO-RADS score was used. The chest CT findings fell into the CO-RADS 4 class, indicating high suspicion of COVID-19 pulmonary involvement [5,6].

**Figure 2:**
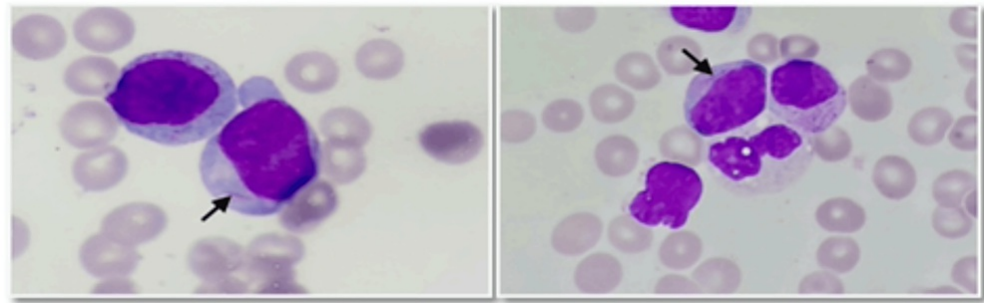
Bone marrow aspirate shows 70% blast infiltration and needle-sharped cytoplasmic inclusions corresponding to Auer bodies (the arrows indicate the requested regions).

Considering these results, a bone marrow aspiration was performed, revealing blast infiltration of 70% granular blasts, which were positive for myeloperoxidase with Auer rods (Figure 2). Based on these findings, the patient was diagnosed with acute myeloblastic leukemia, which was confirmed through additional tests such as immunophenotyping, karyotype, and molecular biology.

The therapeutic approach for the patient centered around various interventions. Initially, oxygen therapy was administered using a high-concentration mask at a rate of 10 liters per minute. Intravenous hyperhydration was provided at a rate of three liters per square meter. The patient received transfusion support, including platelets and fresh frozen plasma. To address the leukocytosis, cytoreductive treatment with hydroxyurea and methylprednisolone was initiated.

Due to an unsatisfactory response to symptomatic treatment after three days, flashes of chemotherapy were administered, involving cytarabine at a dosage of 100 mg on days one and two. Following a pre-therapeutic assessment, which revealed a reduction in interstitial and alveolar infiltration on the chest x-ray, the patient underwent induction treatment using standard-dose cytarabine combined with daunorubicin.

During the course of treatment, the patient developed febrile aplasia and septicemia caused by *Escherichia coli*. The infection responded well to imipenem and amikacin.

**Figure 3:**
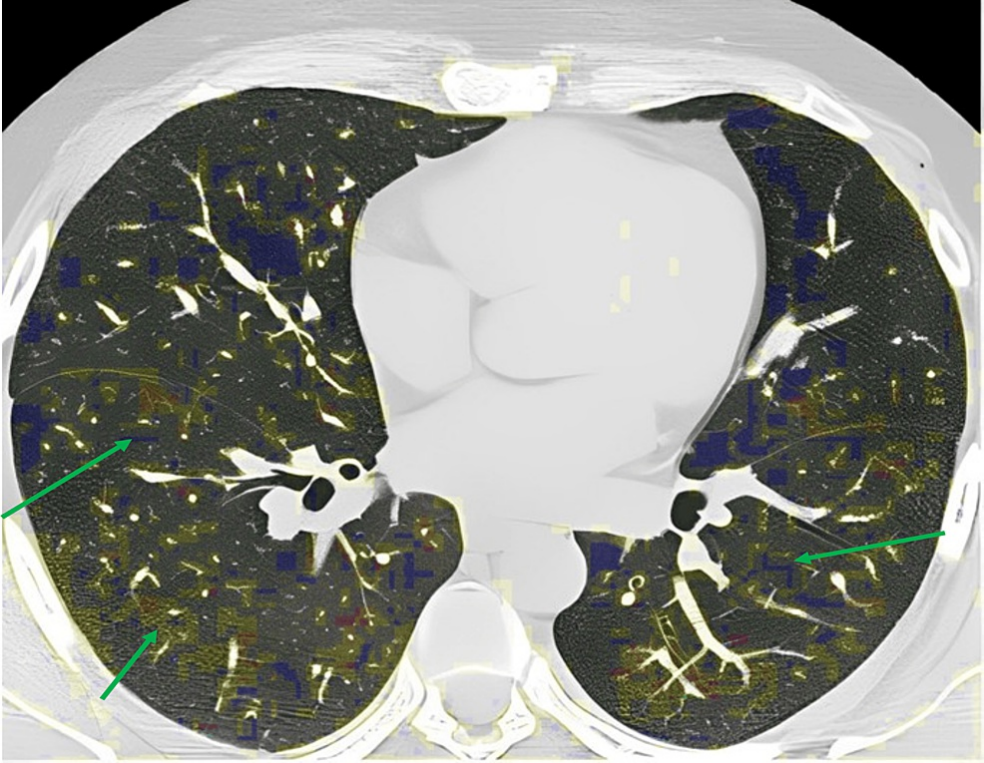
Axial chest CT scan after chemical treatment showed complete radiological clearance with the disappearance of all lung lesions (the arrows indicate the reduction or disappearance of lesion areas after treatment).

On day 23, the patient's aplasia resolved, and a myelogram conducted at the end of the induction treatment showed complete hematological remission. A subsequent chest CT scan demonstrated complete radiological clearance, with all lung lesions disappearing (Figure 3).

## Discussion

Acute myeloid leukemia (AML) is the predominant type of leukemia in adult patients, constituting approximately 80% of all leukemia cases [7]. In untreated AML, there is an abnormal proliferation of leukemia cells, leading to hyperleukocytosis, characterized by a white blood cell count exceeding 100,000/μl, in about 5% to 20% of patients with the disease [2]. This high leukocyte count can result in three main complications: leukemic pulmonary infiltration, lysis pneumopathy, and leukostasis. The latter can be attributed to two distinct mechanisms. The differentiation between these complications relies on various criteria, including clinical, radiological, and anatomopathological factors [1] (Table 2).

**Table 2:** The clinical anatomopathological and radiological criteria. Reference [1].

Criteria	Leukostasis	Leukemic pulmonary infiltration	Lysis pneumopathy
Time of appearance	Before chemotherapy	Before chemotherapy	Immediately until the onset of neutropenia
Hyperleukocytosis	Yes	Not compulsory	Yes or no
Radiological anomalies	Non-specific	Infiltration along lymphatic vessels in interstitial septal, pleural, and peribronchovascular tissues	Sparse and multilobar pneumonia
Anatomopathological results	Occlusion of the vascular lumen by aggregates of blasts	Infiltration of blasts into the pulmonary arteries, bronchi, and bronchioles	Diffuse alveolar lesions

**Figure 4:**
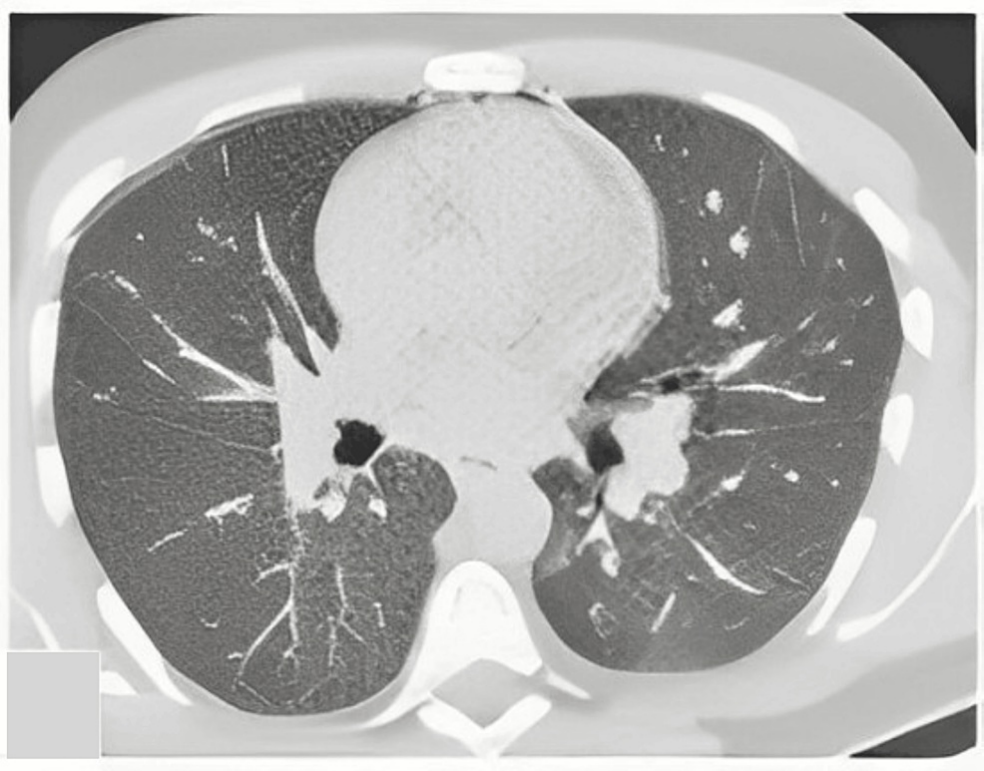
Patient with pulmonary leukostasis. Reference [1].

**Figure 5:**
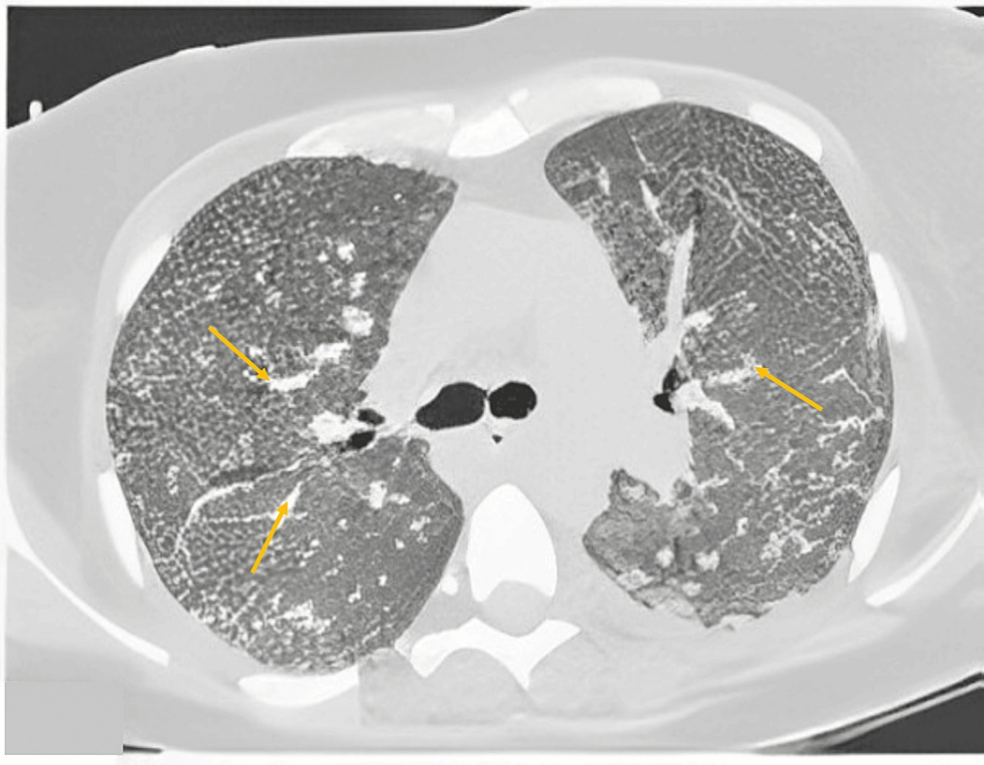
Leukemic pulmonary infiltration. Female patient with leukemic pulmonary infiltration: interstitial syndrome with septal and peribronchovascular thickening (the arrows indicate the requested regions). Reference [1].

**Figure 6:**
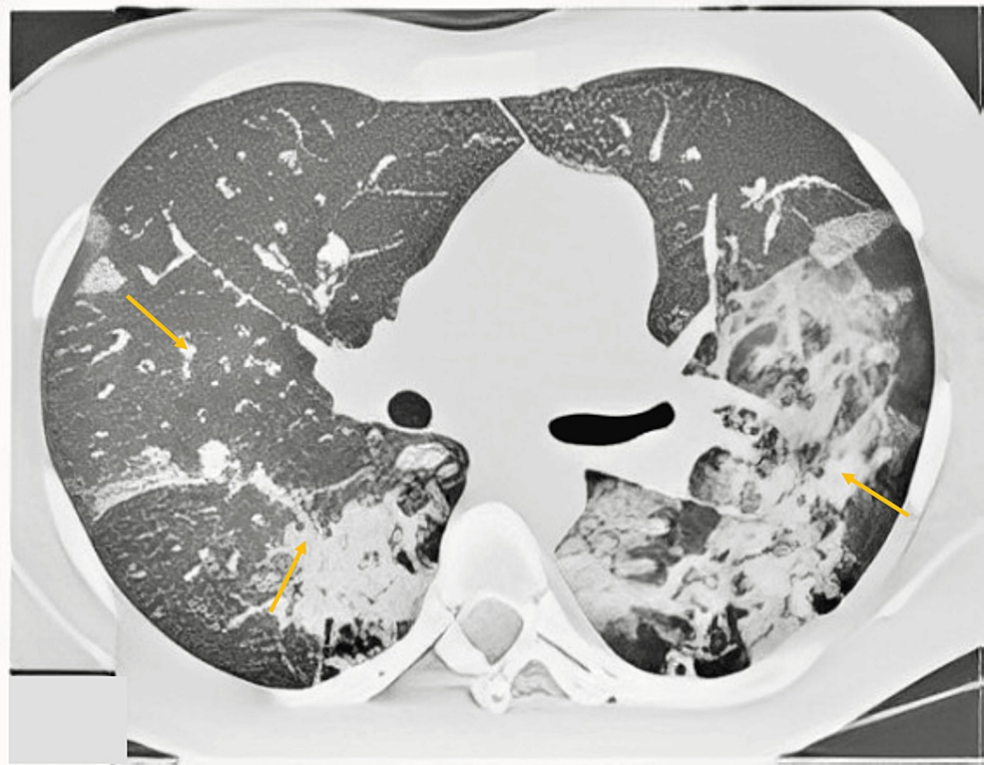
Female patient with lytic pneumopathy: bilateral ground glass opacities corresponding to intra-alveolar hemorrhage (the arrows indicate the requested regions). Reference [1].

Figures 4-6 depict the radiological abnormalities listed in the table above in the respective cases: leukostasis, leukemic pulmonary infiltration, and lysis pneumopathy.

Research has shown that acute hyperleukocytic myeloid leukemia induces an increase in blood viscosity due to the elevated leukocyte count and reduced cell deformability. This increased blood viscosity disrupts blood circulation in the lungs and brain, contributing to leukostasis complications [8]. This phenomenon is one of the reasons for the higher incidence of leukostasis in AML compared to other types of leukemia, such as chronic myeloid leukemia, acute lymphoid leukemia, and chronic lymphoid leukemia. Nonetheless, leukostasis has also been observed in some AML patients without hyperleukocytosis, suggesting the involvement of qualitative mechanisms rather than quantitative mechanisms. Specifically, this is attributed to the interaction between blasts and the vascular endothelium.

**Figure 7:**
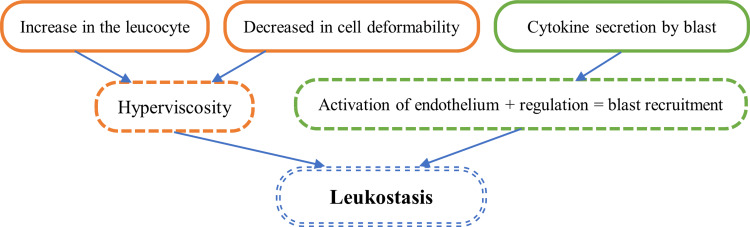
Physiopathology of leukostasis (the arrows indicate implications). References: [1-8].

Studies conducted by Stucki and Ai have revealed that blasts secrete cytokines, namely tumor necrosis factor (TNF) and IL-1b, which activate endothelial cells. This activation, in turn, results in the regulation of adhesion molecules such as selectin and VCAM-1, which leads to the recruitment and adhesion of blast cells to the vascular endothelium [8] (Figure 7). These processes contribute to the development of leukostasis in AML patients, even in the absence of hyperleukocytosis.

Clinically, hyperleukocytosis syndrome, or leukostasis, can lead to significant pulmonary and/or neurological damage, and if diagnosis and treatment are delayed, it may result in up to 40% of deaths within a week [1]. In the brain, leukostasis can manifest as headaches, psychomotor retardation, drowsiness, confusion, and even intracranial hemorrhage [1].

In the lungs, it can cause respiratory distress, which can resemble the symptoms seen in certain pulmonary infections like COVID-19 [9]. Thus, when presented with such cases, it is essential to conduct a comprehensive radiological and biological evaluation to distinguish between infectious pulmonary complications and non-infectious complications that may arise during the course of acute myeloid leukemia (Table 3).

**Table 3:** Comparative table between leukostasis and COVID-19. References: [1-10]. RT-PCR: reverse transcription polymerase chain reaction.

	Leukostasis	COVID-19
Clinical signs	Fever, dyspnea, asthenia splenomegaly, adenopathy, purpura, pale conjunctiva	Fever, cough, myalgia headache, chills, nausea or vomiting, diarrhea, ageusia, and conjunctival congestion
Biological check-up	Hyperleukocytosis >100,000/ul; thrombocytopenia; low hemoglobin	High CRP, high erythrocyte sedimentation rate, lymphopenia
Chest CT scan	Frosted glass opacities	Ground-glass opacity; irregular interlobular septal thickening; air bronchogram; crazy-paving pattern
RT-PCR	Negative	Positive (often)
Antigen test	Negative	Positive

Considering the pathophysiology of leukostasis, prompt treatment is of utmost importance, and the therapeutic strategy should focus on eliminating leukemic blasts through leukapheresis and cytoreduction using chemotherapy or hydroxyurea [11]. Prior to initiating intensive treatment, an eligibility test becomes crucial to avoid potential toxicity during therapy. This evaluation incorporates various parameters, such as comorbidities, age, performance status, cytogenetic and molecular characteristics, cumulative anthracycline exposure, and bone marrow reserve [11]. Eligible patients are typically started on induction treatment following the European LeukemiaNet (ELN)-recommended [3-7] protocol, involving a continuous infusion of cytarabine for seven days and three days of daunorubicin [11].

However, cytoreduction with hydroxyurea (dose ranging from 50 mg/kg/day to 150 mg/kg/day) might take several days, limiting its utility in life-threatening leukostasis cases. In such situations, adding a dose of 10 mg of dexamethasone every six hours to chemotherapy and broad-spectrum antibiotics has shown promising results. Recent studies indicate that dexamethasone reduced the intensive care unit mortality rate by 30% in AML patients with leukostasis without compromising the anti-leukotoxic effect of cytarabine [11].

Cytoreduction treatment rapidly destroys tumor cells, leading to hyperuricemia, hyperphosphatemia, hypocalcemia, and hyperkalemia, characteristic of tumor lysis syndrome. Preventive strategies to manage tumor lysis syndrome include hyperhydration, the introduction of allopurinol or rasburicase, and a recombinant urate oxidase [11]. In select cases, leukapheresis might be beneficial for patients with overt leukostasis syndrome, provided there are no contraindications such as cardiovascular comorbidities, hemodynamic instability, or coagulation disorders, which necessitate careful assessment to minimize procedural risks [12].

**Figure 8:**
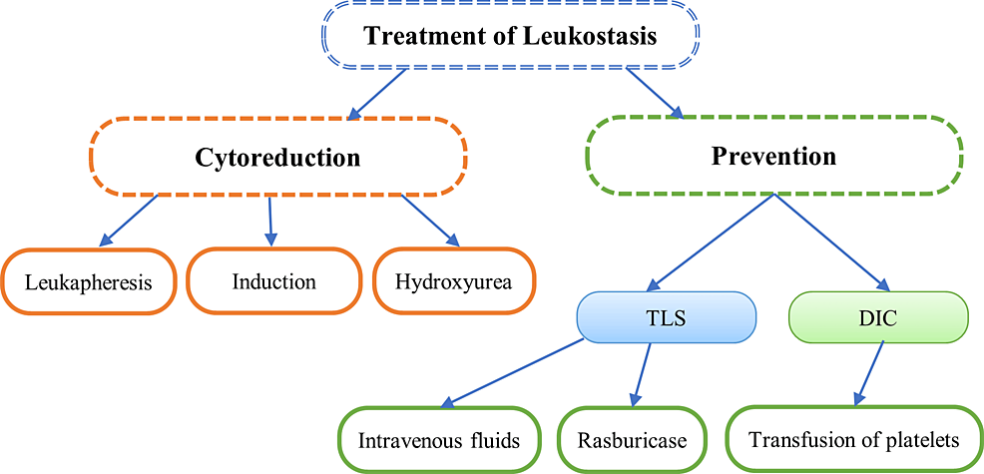
Treatment of leukostasis (the arrows indicate based on). Reference: [11]; TLS: tumor lysis syndrome; DIC: disseminated intravascular coagulation.

Inserting large-bore central venous apheresis catheters may present a challenge, and experienced physicians should perform the procedure to minimize bleeding risks. Disseminated intravascular coagulation, as observed in our case, represents a formal contraindication to leukapheresis [13]. Blood transfusions are temporarily suspended wherever possible until the white blood cell count declines. If necessary, transfusions should be administered slowly. In cases of complete anticoagulation with heparin, prophylactic platelet transfusions are carried out to maintain a platelet count above 20,000 to 30,000/µL or 50,000/µL until the leukocyte count decreases, and the clinical situation stabilizes [12-14]. The entire treatment of leukostasis is summarized in Figure 8.

## Conclusions

Our results highlight the importance of distinguishing between leukostasis and COVID-19 based on an analysis of clinical signs, chest CT scans, RT-PCR, and biological check-ups. The therapeutic approach based on leukapheresis and cytoreduction by chemotherapy or hydroxyurea has shown promising results in reducing the leukemic cell burden and managing leukostasis-related complications. In addition, our research highlights the need for vigilant monitoring and preventive strategies to combat tumor lysis syndrome and optimize blood transfusions in order to minimize potential risks and complications. Collaboration between healthcare professionals, including hematologists, and qualified medical teams is essential to achieve positive results in the management of leukostasis.
